# 590. CLABSI Prevention: Interactive Staff Education Process in a Burn/wound Mixed Acuity Unit

**DOI:** 10.1093/jbcr/irag033.384

**Published:** 2026-04-06

**Authors:** Sierra Turner, Morgan M Lofgren, Denise Mazzacano Searles

**Affiliations:** Legacy Oregon Burn Center, Portland, OR; Legacy Oregon Burn Center, Portland, OR; Legacy Oregon Burn Center, Portland, OR

## Abstract

**Introduction:**

Hospital-acquired infections such as central line-associated bloodstream infections (CLABSI) can increase patient length of stay and hospital costs. Consistent, correct central line care is essential to preventing infection. Nursing turnover and variation in preceptor practice can lead to inconsistent knowledge of central line care and increased infection risk. Periodic staff education is helpful but often takes the form of online self-learning modules. Our Clinical Nurse Educators designed a one-on-one, hands-on learning module to provide an additional learning modality to improve central line skills.

**Methods:**

Hands-on central line training took place on day and night shifts, over a period of three months, with all nurses meeting one-on-one with trainers. To ensure procedural consistency, all trainers received instruction from a nurse educator, utilizing Elsevier-provided instructions. Supporting documents were incorporated to deepen understanding of central line insertion, maintenance, and documentation practices. Key takeaways for each topic were clearly outlined in the training agenda to reinforce learning objectives. Nurses demonstrated proficiency via verbal response and return demonstration. Education covered the following topics:

• nursing expectations during central line insertion.

• central line dressing change.

• unit-specific process for central line dressings over burn/graft.

• tubing/cap change.

• CHG bath.

• Lab and blood cultures.

• documentation.

A 12-point quiz and a 4-question comfort scale were administered prior to the start of the CLABSI education program and again after training was complete. Pre- and post-training results were compared with a Mann–Whitney U test to measure knowledge and comfort level changes.

**Results:**

We found a statistically significant increase in overall assessment scores after training, from 8 to 9 (*p*=.002). The greatest increase was seen in nurses with <5 years’ experience. We also saw an increase in mean comfort scale scores. As with the quiz scores, the greatest changes were seen in less experienced RNs.

**Conclusions:**

Hands-on, one-on-one training was beneficial, leading to an increase in knowledge of central line care and CLABSI prevention as well as increasing confidence around central line care. As newer nurses make up a large proportion of our staff, seeing greater changes in that subset of nurses is promising.

**Applicability of Research to Practice:**

Our staff receive recurring education on multiple skills, often in the form of online learning modules or in a group skills day setting. Modules such as this, with hands-on, one-on-one training, can be a useful adjunct to other teaching methods.

**Funding for the study:**

N/A.

